# Transcriptome Profiling of the Whitefly *Bemisia tabaci* MED in Response to Single Infection of *Tomato yellow leaf curl virus, Tomato chlorosis virus*, and Their Co-infection

**DOI:** 10.3389/fphys.2019.00302

**Published:** 2019-04-03

**Authors:** Tian-Bo Ding, Jie Li, Er-Hu Chen, Jin-Zhi Niu, Dong Chu

**Affiliations:** ^1^Key Laboratory of Integrated Crop Pest Management of Shandong Province, College of Plant Health and Medicine, Qingdao Agricultural University, Qingdao, China; ^2^Key Laboratory of Entomology and Pest Control Engineering, College of Plant Protection, Southwest University, Chongqing, China

**Keywords:** *Bemisia tabaci*, *Tomato yellow leaf curl virus*, *Tomato chlorosis virus*, co-infection, transcriptome

## Abstract

*Tomato yellow leaf curl virus* (TYLCV) and *Tomato chlorosis virus* (ToCV) are two of the most devastating cultivated tomato viruses, causing significant crop losses worldwide. As the vector of both TYLCV and ToCV, the whitefly *Bemisia tabaci* Mediterranean (MED) is mainly responsible for the rapid spread and mixed infection of TYLCV and ToCV in China. However, little is known concerning *B. tabaci* MED's molecular response to TYLCV and ToCV infection or their co-infection. We determined the transcriptional responses of the whitefly MED to TYLCV infection, ToCV infection, and TYLCV&ToCV co-infection using Illumina sequencing. In all, 78, 221, and 60 differentially expressed genes (DEGs) were identified in TYLCV-infected, ToCV-infected, and TYLCV&ToCV co-infected whiteflies, respectively, compared with non-viruliferous whiteflies. Differentially regulated genes were sorted according to their roles in detoxification, stress response, immune response, transport, primary metabolism, cell function, and total fitness in whiteflies after feeding on virus-infected tomato plants. Alterations in the transcription profiles of genes involved in transport and energy metabolism occurred between TYLCV&ToCV co-infection and single infection with TYLCV or ToCV; this may be associated with the adaptation of the insect vector upon co-infection of the two viruses. Gene Ontology (GO) and Kyoto Encyclopedia of Genes and Genomes (KEGG) pathway enrichment analyses demonstrated that the single infection with TYLCV or ToCV and the TYLCV&ToCV co-infection could perturb metabolic processes and metabolic pathways. Taken together, our results provide basis for further exploration of the molecular mechanisms of the response to TYLCV, ToCV single infection, and TYLCV&ToCV co-infection in *B. tabaci* MED, which will add to our knowledge of the interactions between plant viruses and insect vectors.

## Introduction

More than 75% of plant viruses are transmitted by insect vectors, most of which belong to hemipteran (suborder Homoptera) families, such as whiteflies, aphids, and planthoppers (Hogenhout et al., 2008). *Bemisia tabaci* (Gennadius) (Hemiptera: Aleyrodidae), commonly known as the sweetpotato whitefly, has caused tremendous damage to the tomato crops in greenhouses and fields worldwide (Valverde et al., 2004). *B. tabaci* is a cryptic species complex consisting of at least 39 cryptic species (Alemandri et al., 2015), among which the Middle East-Asia Minor (MEAM1) (formerly referred to as biotype B) and the Mediterranean (MED) (formerly referred to as biotype Q) species have become the most destructive alien species in many regions of the world (De Barro et al., 2011). The whitefly MED was first detected in China in 2003 (Chu et al., 2006), and has gradually displaced MEAM1 and become the dominant cryptic species of *B. tabaci* in China (Pan et al., 2011; Rao et al., 2011). The whitefly is an effective vector of numerous plant viruses, the majority of which are Begomoviruses. Several viruses belonging to other genera, including *Crinivirus, Ipomovirus, Torradovirus*, and *Carlavirus*, can also be transmitted by whiteflies (Navas-Castillo et al., 2011; Polston et al., 2014).

*Tomato yellow leaf curl virus* (TYLCV) (Geminiviridae), the type member of the genus *Begomovirus*, is one of the most devastating viruses of cultivated tomato worldwide; the virus is transmitted by *B. tabaci* in a persistent circulative manner (Czosnek, 2007; Hogenhout et al., 2008). In China, TYLCV was first detected in 2006 in Shanghai (Wu et al., 2006), and has rapidly spread to many provinces, including Zhejiang, Jiangsu, Shandong, Hebei, and Beijing (Ji et al., 2008; Mugiira et al., 2008; Sun et al., 2009; Zhang et al., 2010; Zhou et al., 2010; Pan et al., 2012). A previous study has confirmed that *B. tabaci* MEAM1 and MED contributed to the TYLCV epidemic across China, and that the introduction of MED accelerated the prevalence of TYLCV (Pan et al., 2012). Recently, another devastating tomato virus, *Tomato chlorosis virus* (Closteroviridae: *Crinivirus*), has reached an outbreak level in several areas of the Chinese mainland, including Beijing, Tianjin, Shandong, Henan, Jiangsu, Neimenggu, and Guangdong (Zhao et al., 2013; Liu et al., 2014; Gao et al., 2015; Hu et al., 2015; Wu et al., 2016; Zheng et al., 2016; Tang et al., 2017; Wei et al., 2018), resulting in significant crop losses. ToCV is uniquely transmitted in a semi-persistent mode by two distinct whitefly genera, *Bemisia* and *Trialeurodes*, viz. *B. tabaci, T. abutilonea*, and *T. vaporariorum* (Wisler et al., 1998; Wintermantel and Wisler, 2006). Furthermore, previous research suggests that the rapid spread of ToCV in China was associated with the transmission by MED (Dai et al., 2016; Shi et al., 2018). Notably, the co-infection of TYLCV and ToCV have been detected in several regions of China, including Shandong and Jiangsu Provinces (Zhao et al., 2014; Wu et al., 2016). Following the confirmation of the transmission of TYLCV and ToCV by *B. tabaci* MED in China, we speculated that the TYLCV&ToCV co-infection in the field was probably due to the spread of *B. tabaci* MED.

Interactions between plant viruses and insect vectors are important for both the dispersal of the plant viruses and the population dynamics of the insect (Stout et al., 2006). Plant viruses can have direct or indirect impacts on insect vectors. For example, two Begomoviruses [*Tabacco curly shoot virus* (TbCSV) and *Tomato yellow leaf curl China virus* (TYLCCNV)] infecting tobacco plants significantly increased the fecundity and longevity of their insect vector *B. tabaci* MEAM1 (Jiu et al., 2007). In addition, TYLCV infection also benefitted its vector *B. tabaci* MED by improving the growth, survival, and reproduction (Su et al., 2015). However, ToCV infection decreased the performance of *B. tabaci* MED on tomato plants as measured by declines in longevity and fecundity (Li et al., 2018).

Next-generation sequencing has been shown to be an efficient means of examining the interaction mechanisms between plant viruses and insects. Many transcriptome studies have been performed to analyze the responses to diverse viral infections in whiteflies. When *B. tabaci* MEAM1 was infected with TYLCCNV, the immune responses were activated, and detoxification activity and energy costs were simultaneously attenuated (Luan et al., 2011, 2013). Early studies also revealed that a number of genes involved in transport, binding, metabolism, signal transduction, receptors and lysosomes were differentially regulated when *B. tabaci* MEAM1 fed on TYLCV- and ToCV-infected tomato plants (Kaur et al., 2017; Hasegawa et al., 2018). However, previous viral transcriptome studies were mainly performed on *B. tabaci* MEAM1, and the focus was limited to the response to a single virus infection. Little is known concerning how *B. tabaci* MED responds to single TYLCV and ToCV infection. Moreover, the molecular response of *B. tabaci* to the co-infection with TYCLV and ToCV remains unknown.

In this study, we compared the transcriptional responses in *B. tabaci* MED after feeding on TYLCV- and ToCV-infected, TYLCV&ToCV co-infected and uninfected tomato plants during a 24-h acquisition access period (AAP), respectively. A number of genes involved in defense response, transport, primary metabolism, cell function, and fitness responded to viral infection. We also used the Gene Ontology (GO) and Kyoto Encyclopedia of Genes and Genomes (KEGG) databases to further annotate the functions of the differentially expressed genes (DEGs). Additionally, we compared the similarities and differences between MED whiteflies infected by a single virus (TYLCV or ToCV) and whiteflies co-infected by TYLCV&ToCV. The results provide a comprehensive view of the molecular response to diverse forms of viral infection in the MED whitefly and yield new insights into the interactions between insect vectors and multiple viruses. To our knowledge, this is the first study to analyze the transcriptional changes in response to viral co-infection in whitefly vectors.

## Materials and Methods

### Insect Culture and Virus Source

A colony of *B. tabaci* MED originating in 2012 from Ji'nan, Shandong, China has been maintained on cotton plants (*Gossypium hirsutum* L. cv. Lu-Mian 21), a non-host for TYLCV and ToCV for 6 years. The TYLCV-infected, ToCV-infected, and TYLCV&ToCV co-infected tomato plants (*Solanum lycopersicum* M) were collected from Qingdao, Shandong, China in 2014, and the viruses were maintained using tomato plants (*S. lycopersicum* M. cv. Zhongza 9) via whitefly transmission as described previously (Li et al., 2018). Both the whiteflies and the plants were cultured in separate climate chambers at 27°C, 60% RH, and a 16:8 (L: D) of photoperiod. The *B. tabaci* MED population was confirmed using the *Vsp* I-based mtCOI-RFLP method (Chu et al., 2012).

### Virus Acquisition and Sample Collection

To obtain the viruliferous and non-viruliferous whiteflies, 2,400 female adults were collected and transferred onto TYLCV-infected, ToCV-infected, TYLCV&ToCV co-infected, and uninfected tomato plants for 24 h. Approximately 200 live whiteflies were collected from virus-infected or uninfected tomato plants following a 24-h AAP for each of the two biological replications. The whiteflies were frozen in liquid nitrogen and stored at −80°C. To confirm virus acquisition and determine the efficiency of acquisition for each virus, DNA was extracted from 15 individuals fed on TYLCV-infected and TYLCV&ToCV co-infected tomato plants using a TIANamp Micro DNA Kit (TIANGEN, China). Total RNA was extracted from 15 individuals fed on ToCV-infected and TYLCV&ToCV co-infected tomato plants using TRIzol Reagent (Thermo Fisher, USA). The first-strand cDNA was synthesized following the procedures for the PrimerScript RT Reagent Kit (Perfect real-time) (TaKaRa, Japan). PCR analyses for the detection of TYLCV and ToCV were conducted using Golden Star T6 Super PCR Mix (TsingKe, China) with the primers TYLCV-F/R (Li et al., 2012) and ToCV-F/R (Dovas et al., 2002), respectively (Supplementary Table S1). The individuals fed on the uninfected tomato plants served as the negative control.

### cDNA Library Preparation and Sequencing

Total RNA was extracted separately from viruliferous and non-viruliferous whiteflies using TRIzol Reagent (Thermo Fisher, USA) according to the manufacturer's protocol. RNA quality and concentration were verified using 1% agarose gels, a NanoPhotometer® spectrophotometer (IMPLEN, USA), and the Qubit RNA Assay Kit in a Qubit® 2.0 Fluorometer (Life Technologies, USA). The integrity of total RNA was determined using the RNA Nano 6000 Assay Kit of the Bioanalyzer 2100 system (Agilent Technologies, USA).

The cDNA libraries were generated from 1.5 μg RNA of each sample using NEBNext® Ultra^TM^ RNA Library Prep Kit for Illumina (NEB, USA) following the manufacturer's instructions, and the index codes were added to attribute sequences to each sample. The quality of the libraries was evaluated using the Agilent Bioanalyzer 2100 system. The cDNA libraries were sequenced for 125/150 bp paired-end reads on an Illumina HiSeq Xten platform (Novogene Bioinformatics Technology Co. Ltd, China).

### Transcriptome Assembly and Differential Expression Analysis

In order to obtain clean data, the raw reads were cleaned by removing any reads containing adapter, reads containing poly-N, and low quality reads. The genomes of *B. tabaci* MED (Xie et al., 2017) and MEAM1 (Chen et al., 2016) were used for alignment of the clean reads. Indices of the reference genomes were built using Bowtie v2.2.3 (Langmead and Salzberg, 2012), and the high-quality paired-ended clean reads were aligned to the reference genome using HISAT 2.0.4 (Kim et al., 2015). The reads numbers mapped to each gene were counted using HTSeq v0.6.1. The FPKM (expected number of Fragments Per Kilobase of transcript sequence per Million base pairs sequenced) of each gene was calculated based on the length of the gene and the reads count mapped to that gene. Differential expression analysis of viruliferous and non-viruliferous whiteflies was performed using the DESeq2 R package (1.0) (Love et al., 2014). The resulting *P*-values were adjusted using Benjamini and Hochberg's approach for controlling the false discovery rate (FDR) (Benjamini and Hochberg, 1995). Genes with an adjusted *P*-value (*q*-value) < 0.05 found by DESeq were assigned as differentially expressed. The identification of genes related to viruses infection was conducted based on the gene annotation and differential expression analysis.

### GO and KEGG Pathway Analysis

GO enrichment analysis of the differential expression of genes across the samples was carried out using the GOseq R package (Young et al., 2010). Additionally, the statistical enrichment of the differential expression genes in KEGG pathways was implemented by the KOBAS software.

### RT-qPCR Validation

To validate the DEG analysis results, the expression profiles of 11 genes were measured by RT-qPCR with SDHA as the internal control gene (Li et al., 2013). Primers for RT-qPCR were designed using the Primer 3.0 software (http://bioinfo.ut.ee/primer3-0.4.0/) and are listed in Supplementary Table S1. All of the whitefly samples for the RT-qPCR validation were prepared according to the method described above. Each treatment contained three biological replications. The same total RNA extraction and cDNA synthase methods were used as described above for the detection of ToCV in whiteflies. RT-qPCR assays were performed in 20 μL using the SYBR Premix Ex Taq^TM^ II (Perfect Real Time) (TaKaRa, Dalian, China) according to the manufacturer's instructions. The reactions were conducted using a qTower 2.2 real-time PCR Thermal Cycler (Analytikjena, Germany) under the following conditions: 95°C for 2 min; 40 cycles of 95°C for 15 s and 60°C for 30 s; melting curve generation (60°C to 95°C). The relative expression ratios were calculated using the 2^−ΔΔ*CT*^ method (Livak and Schmittgen, 2001).

## Results

### Virus Infection Status of Whiteflies Selected for RNA-Seq

In order to make sure the basis of comparison between virus-infected and non-viruliferous colonies of *B. tabaci* MED, the infection rates were estimated for the whiteflies feeding on virus-infected tomato plants for 24 h. Both the infection rates of TYLCV and ToCV reached 100% in the whiteflies after a 24-h AAP feeding on TYLCV and ToCV singly infected tomato plants (Supplementary Figure S1). However, the lower infection rates of TYLCV (93.33%) and ToCV (80%) were observed in the whiteflies feeding on TYLCV&ToCV co-infection plants (Supplementary Figure S1).

### Overview of Illumina Sequencing and Transcriptome Assembly

To determine the transcriptome profiles of *B. tabaci* MED in response to TYLCV infection, ToCV infection, and TYLCV&ToCV co-infection, we performed RNA-seq analysis of female adults that had fed on the respective tomato plants for 24 h Eight cDNA libraries of viruliferous and non-viruliferous whiteflies were sequenced, generating 30,475,776 and 43,101,948 raw reads, respectively (Table 1). After cleaning and quality checks, 29,813,838 to 41,978,676 clean reads were obtained and mapped to the whitefly (*B. tabaci* MED) reference genome, for the mapping rates of 80.85 to 82.36% (Table 1). Additionally, Pearson's correlation analysis indicated that the two replicated libraries of each treatment were highly correlated (Pearson's *r* = 0.958–0.968) (Supplementary Figure S2).

**Table 1:** Summary statistics of RNA-Seq libraries from *Bemisia tabaci* MED fed for 24 h on virus-infected or uninfected tomato plants.

**Sample^***a***^**	**Raw reads number**	**Clean reads number**	**Clean reads rate (%)**	**Mapped to genome**	
				**Mapped number**	**Mapping rate (%)**
TYLCV-1	32,076,630	31,266,388	97.47	25,731,071	82.30
TYLCV-2	34,205,824	33,444,404	97.77	27,497,666	82.22
ToCV-1	32,752,142	31,958,512	97.58	26,320,948	82.36
ToCV-2	31,876,568	31,182,608	97.82	25,499,858	81.78
TYLCV&ToCV-1	43,101,948	41,978,676	97.39	34,195,336	81.46
TYLCV&ToCV-2	32,569,312	31,723,612	97.4	25,648,753	80.85
NV-1	30,475,776	29,813,838	97.83	24,387,996	81.80
NV-2	34,931,068	34,065,894	97.52	27,934,857	82.00

a TYLCV: TYLCV-infected whiteflies; ToCV: ToCV-infected whiteflies; TYLCV&ToCV: TYLCV&ToCV co-infected whiteflies; NV: uninfected (non-viruliferous) whiteflies

### Global Patterns of Gene Expression in Response to Different Patterns of Viral Infection

**Figure 1:**
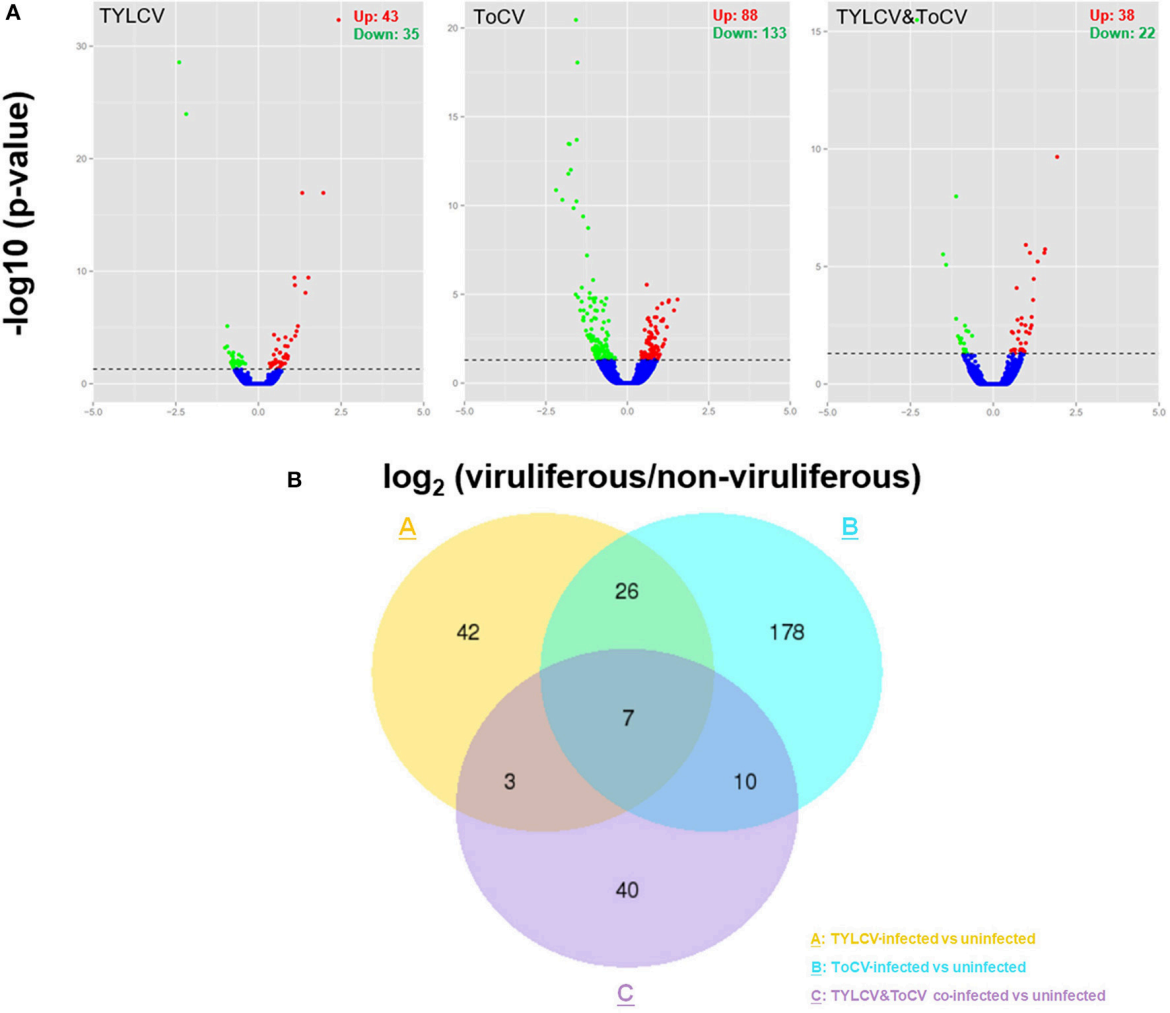
Differentially expressed genes (DEGs) in *Bemisia tabaci* MED in response to TYLCV infection, ToCV infection, and TYLCV&ToCV co-infection. **(A)** Volcano plots of differentially expressed genes in whiteflies infected with TYLCV, ToCV, and TYLCV&ToCV compared to non-viruliferous whiteflies. Dots above the horizontal dotted line indicate the DEGs with an adjusted *P*-value < 0.05. Red dots denote significantly upregulated genes, while green dots denote significantly downregulated genes. **(B)** Venn diagram depicting unique and common DEGs in whiteflies in response to TYLCV infection, ToCV infection, and TYLCV&ToCV co-infection.

A total of 359 genes were differentially expressed in whiteflies feeding on TYLCV-infected, ToCV-infected, and TYLCV&ToCV co-infected tomato plants compared to whiteflies that fed on uninfected tomato plants (Figure 1A). In TYLCV and ToCV infected whiteflies, 78 (43 upregulated, 35 downregulated) and 221 (88 upregulated, 133 downregulated) DEGs were detected, while only 60 (38 upregulated, 22 downregulated) genes were differentially expressed due to TYLCV&ToCV co-infection (Figure 1A).

Although most of the DEGs in response to different patterns of viral infection were diverse, there were seven common genes differentially expressed in whiteflies feeding on TYLCV-infected, ToCV-infected, and TYLCV&ToCV co-infected tomato plants (Figure 1B). Totals of 42.31% and 14.93% of the virus-responsive genes were shared between TYLCV- and ToCV-infected whiteflies. Compared to non-viruliferous whiteflies, only ten DEGs were in common between TYLCV-infected and TYLCV&ToCV co-infected whiteflies. However, greater proportions of common genes altered by ToCV (7.69%) and TYLCV&ToCV (28.33%) were identified (Figure 1B).

### Differentially Regulated Genes Associated With Detoxification

Compared to non-viruliferous whiteflies, three differentially regulated cytochrome P450 (P450) genes involved in detoxification were identified in TYLCV-infected whiteflies. One P450 gene was significantly upregulated, while the other two P450 genes were significantly downregulated (Table 2). We found a total of 12 DEGs encoding detoxification enzymes in ToCV-infected whiteflies, including five P450s (one upregulated, four downregulated), a downregulated carboxylesterase (CarE), four downregulated UDP-glucuronosyltransferases (UGTs), and two upregulated ATP-binding cassette transporters (ABCs) (Table 2). Only one P450 gene, *BTA015105.1*, was identified and downregulated in response to TYLCV&ToCV co-infection (Table 2); the same gene was also detected in TYLCV and ToCV singly infected whiteflies with the same expression pattern.

**Table 2:** Differentially regulated genes associated with detoxification.

**Virus infection**	**Gene ID**	**Annotation**	**V^***a***^**	**NV^***b***^**	**FC^***c***^**	**q-value**	**Direction**	**Gene ID-B^***d***^**
TYLCV	BTA009037.1	Cytochrome P450	770.83	458.24	0.65	0.0018	Up	Bta07286
	BTA025848.1	Cytochrome P450	1563.21	2287.27	−0.50	0.0042	Down	Bta07221
	BTA015105.1	Cytochrome P450	1861.63	2688.60	−0.48	0.0104	Down	Bta08018
ToCV	BTA009039.1	Cytochrome P450	337.76	163.53	0.82	0.0254	Up	Bta07284
	BTA015105.1	Cytochrome P450	805.62	2640.14	−1.58	3.44E−21	Down	Bta08018
	BTA025209.1	Cytochrome P450	115.10	290.08	−0.95	0.0147	Down	Bta02801
	BTA025872.1	Cytochrome P450	2350.89	3801.35	−0.63	0.0085	Down	Bta05554
	BTA025848.1	Cytochrome P450	1450.12	2246.02	−0.60	0.0020	Down	Bta07221
	BTA010352.1	Carboxylesterase	641.86	1020.42	−0.61	0.0147	Down	Bta08899
	BTA016010.1	UDP-glucuronosyltransferase	38.88	125.80	−1.03	0.0146	Down	Bta06665
	BTA011174.1	UDP-glucuronosyltransferase	13.30	123.24	−0.94	0.0263	Down	Bta02603
	BTA018877.1	UDP-glucuronosyltransferase	226.41	407.25	−0.73	0.0106	Down	Bta02228
	BTA013275.1	UDP-glucuronosyltransferase	594.47	919.64	−0.58	0.0201	Down	Bta01304
	BTA029281.1	ATP-binding cassette sub-family G member 1	129.47	36.42	0.97	0.0327	Up	Bta07822
	BTA007131.3	ATP-binding cassette sub-family G member 4	1381.80	836.57	0.67	0.0014	Up	Bta09316
TYLCV&ToCV	BTA015105.1	Cytochrome P450	1744.82	2849.69	−0.64	0.0087	Down	Bta08018

a *Read count values from virus-infected whiteflies*.

b *Read count values from non-viruliferous whiteflies*.

c *Fold change (log2 ratio) of gene expression*.

d *Mapped genes in Bemisia tabaci MEAM1 genome*.

### Differentially Regulated Genes Associated With Stress and Immune Response

A majority of stress response related genes were upregulated in TYLCV-infected whiteflies; these included two alpha-crystallin B chain-like genes, a heat shock protein 70 (HSP70), and a heat shock protein 90 (HSP90) (Table 3). While the gene *BTA014707.1*, which encodes superoxide dismutase (SOD), was significantly downregulated (Table 3). In accordance with TYLCV-infected whiteflies, four of five stress responsive genes were upregulated in whiteflies feeding on ToCV-infected tomato plants, including an HSP70, a Gulia lazarillo, a peroxidase, and a glutathione peroxidase (Table 3). The co-infection with TYLCV and ToCV could also significantly depress the transcription level of the SOD gene *BTA014707.1* (Table 3).

**Table 3:** Differentially regulated genes associated with stress and immune response.

**Virus infection**	**Gene ID**	**Annotation**	**V^***a***^**	**NV^***b***^**	**FC^***c***^**	***q*-value**	**Direction**	**Gene ID-B^***d***^**
**STRESS RESPONSE**								
TYLCV	BTA029550.1	Alpha-crystallin B chain-like	141.75	44.63	0.88	0.0060	Up	Bta03915
	BTA005970.1	Alpha-crystallin B chain-like	1749.54	883.32	0.82	7.61E−05	Up	Bta14756
	BTA025691.1	Heat shock 70 kDa protein	1494.46	877.45	0.66	0.0017	Up	Bta15531
	BTA010369.1	Heat shock protein 90	50110.85	35537.34	0.47	4.61E−05	Up	Bta01899
	BTA014707.1	Superoxide dismutase [Cu-Zn]	30.26	466.43	−2.40	2.70E−29	Down	Bta10955
ToCV	BTA025691.1	Heat shock 70 kDa protein	1549.02	861.63	0.73	0.0137	Up	Bta15531
	BTA019619.1	Glial Lazarillo	411.82	221.36	0.74	0.0257	Up	Bta06480
	BTA015175.2	Peroxidase	4338.36	2626.00	0.66	0.0026	Up	Bta02200
	BTA020045.1	Glutathione peroxidase	1817.27	1259.68	0.49	0.0441	Up	Bta00070
	BTA018068.2	Gamma-interferon-inducible lysosomal thiol reductase putative	1270.66	2035.76	−0.63	0.0014	Down	Bta01175
TYLCV&ToCV	BTA014707.1	Superoxide dismutase [Cu-Zn]	32.82	494.34	−2.30	3.19E−16	Down	Bta10955
**IMMUNE RESPONSE**								
TYLCV	BTA027235.2	Sequestosome-1	5622.29	3545.15	0.61	0.0001	Up	Bta11513
	BTA007837.2	Scavenger receptor class B member, putative	98.53	242.91	−0.89	0.0017	Down	Bta10257
	BTA015120.1	Cathepsin B	166.31	60.50	0.81	0.0160	Up	Bta08035
	BTA028172.1	Cathepsin F	71.19	165.91	−0.76	0.0251	Down	Bta20004
	BTA016813.2	Cathepsin B	2848.14	4454.23	−0.56	0.0135	Down	Bta03880
ToCV	BTA026427.4	Prophenoloxidase subunit 2	874.27	504.16	0.73	0.0005	Up	Bta15615
	BTA008155.2	Serpin	1544.38	1039.13	0.52	0.0476	Up	Bta12484
	BTA021911.1	Ferritin	1557.21	1063.45	0.51	0.0262	Up	Bta07622
	BTA028748.2	Hemocyanin subunit, putative	45284.19	72407.11	−0.65	1.68E−05	Down	Bta12158
	BTA000144.1	Cathepsin B	991.74	3347.14	−1.55	2.03E−14	Down	Bta08697
	BTA016813.2	Cathepsin B	1487.95	4374.04	−1.36	4.10E−10	Down	Bta03880
	BTA028172.1	Cathepsin F	36.32	162.92	−1.34	0.0002	Down	Bta20004
	BTA001253.1	Cathepsin F-like protease	513.12	975.17	−0.78	0.0125	Down	Bta02143
	BTA024606.1	Cathepsin F	8789.26	13205.92	−0.54	0.0429	Down	Bta11871
TYLCV&ToCV	BTA015120.1	Cathepsin B	289.98	64.13	1.09	0.0070	Up	Bta08035
	BTA022401.1	Cathepsin B	5378.46	3428.27	0.60	0.0066	Up	Bta14750

a *Read count values from virus-infected whiteflies*.

b *Read count values from non-viruliferous whiteflies*.

c *Fold change (log2 ratio) of gene expression*.

d *Mapped genes in Bemisia tabaci MEAM1 genome*.

Five DEGs related to the immune response were identified in TYLCV-infected whiteflies, including one upregulated sequestosome-1, one downregulated scavenger receptor, and three differentially expressed genes encoding the cathepsins (one upregulated and two downregulated) (Table 3). Additionally, nine immune related genes were differentially expressed in whiteflies when infected with ToCV (Table 3). The genes encoding prophenoloxidase subunit 2, serpin, and ferritin were upregulated, while one hemocyanin subunit gene was downregulated (Table 3). Five genes associated with the lysosome, including two cathepsin B genes and three cathepsin F genes, were all downregulated (Table 3). Only two cathepsin B genes associated with the lysosome were classified as being associated with the immune response in TYLCV&ToCV co-infected whiteflies, and both were upregulated (Table 3).

### Differentially Regulated Genes Associated With Transport

Four genes associated with transport were all downregulated in TYLCV-infected whiteflies compared with non-viruliferous whiteflies (Table 4). A total of 18 DEGs implicated in transport were identified in ToCV-infected whiteflies, and the expression levels of 14 genes were decreased (Table 4). Among these downregulated genes, five genes (*BTA02849.1, BTA029270.2, BTA019411.1, BTA024233.1*, and *BTA016669.3*) were identified as glucose transporters (Table 4). However, among the DEGs in TYLCV&ToCV co-infected whiteflies, four genes related to transport were all upregulated (Table 4).

**Table 4:** Differentially regulated genes associated with transport.

**Virus infection**	**Gene ID**	**Annotation**	**V^***a***^**	**NV^***b***^**	**FC^***c***^**	***q*-value**	**Direction**	**Gene ID-B^***d***^**
TYLCV	BTA001187.1	Transmembrane protein	43.22	180.38	−1.01	0.0006	Down	Bta09390
	BTA019353.1	Choline transporter-like protein 2	96.99	223.71	−0.80	0.0110	Down	Bta08036
	BTA011410.1	Transporter, putative	361.41	591.09	−0.59	0.0248	Down	Bta13176
	BTA007080.3	Vesicular glutamate transporter 3	995.02	1545.42	−0.56	0.0104	Down	Bta07710
ToCV	BTA029391.1	Annexin	753.13	287.07	1.11	0.0003	Up	Bta06534
	BTA001779.2	Sulfate anion transporter 1	463.49	231.99	0.85	0.0029	Up	Bta12218
	BTA005197.1	Solute carrier family 22 member 4	821.61	485.67	0.68	0.0066	Up	Bta09537
	BTA006299.2	Sodium/nucleoside cotransporter 1	665.38	439.55	0.54	0.0483	Up	Bta01801
	BTA007148.1	Solute carrier family 12 member 2	81.78	186.88	−0.91	0.0135	Down	Bta02878
	BTA028491.1	Facilitated glucose transporter protein 1	64.97	266.52	−1.40	2.63E−05	Down	Bta00944
	BTA029270.2	Facilitated glucose transporter protein 1	206.08	444.35	−0.91	0.0035	Down	Bta11822
	BTA019411.1	Solute carrier family 2, facilitated glucose transporter member 8	17.88	94.84	−1.34	0.0003	Down	Bta09677
	BTA024223.1	Solute carrier family 2, facilitated glucose transporter member 8	203.47	470.58	−1.01	0.0002	Down	Bta02936
	BTA016669.3	Solute carrier family 2, facilitated glucose transporter member 8	42.95	128.37	−1.00	0.0164	Down	Bta08290
	BTA019353.1	Choline transporter-like protein 2	60.80	219.68	−1.40	4.24E−06	Down	Bta08036
	BTA007080.3	Vesicular glutamate transporter 3	565.54	1517.51	−1.24	6.54E−08	Down	Bta07710
	BTA007961.1	Sugar transporter 12	88.99	278.56	−1.23	0.0001	Down	Bta08137
	BTA011410.1	Transporter, putative	221.52	580.43	−1.05	0.0023	Down	Bta13176
	BTA026936.3	Transporter, putative	200.83	450.19	−1.01	2.76E−05	Down	Bta01592
	BTA019415.2	Transporter, putative	115.00	263.92	−0.95	0.0036	Down	Bta09672
	BTA027285.1	Transporter, putative	59.75	145.36	−0.87	0.0474	Down	Bta15790
	BTA008826.1	Protein transport protein Sec61 subunit alpha isoform 2	756.83	1246.58	−0.64	0.0149	Down	Bta10727
TYLCV&ToCV	BTA017507.1	Protein transport protein Sec23A, putative	89.38	24.54	0.94	0.0411	Up	Bta07971
	BTA024080.1	Facilitated trehalose transporter Tret1	3260.64	1562.61	0.83	0.0186	Up	Bta07748
	BTA006299.2	Sodium/nucleoside cotransporter 1	878.09	474.44	0.76	0.0058	Up	Bta01801
	BTA009195.1	Proton-coupled amino acid transporter 1	7601.78	4484.92	0.71	8.28E−05	Up	Bta01722

a *Read count values from virus-infected whiteflies*.

b *Read count values from non-viruliferous whiteflies*.

c *Fold change (log2 ratio) of gene expression*.

d *Mapped genes in Bemisia tabaci MEAM1 genome*.

### Differentially Regulated Genes Associated With Energy Metabolism, Lipid Metabolism, and Protein Synthesis and Amino Acid Metabolism

After feeding on TYLCV-infected tomato plants, only one gene that was considered to be involved in energy metabolism, annotated as ATP synthase gamma chain, was significantly downregulated in whiteflies (Table 5). Six genes involved in carbohydrate metabolism and three genes involved in ATP metabolism were differentially expressed in ToCV-infected whiteflies (Table 5). Moreover, four genes (*BTA020543.1, BTA021845.1, BTA020828.1*, and *BTA001223.1*) associated with carbohydrate metabolism and two genes (*BTA029804.1* and *BTA020850.1*) associated with ATP metabolism appeared among the DEGs in TYLCV&ToCV co-infected whiteflies (Table 5).

**Table 5:** Differentially regulated genes associated with energy metabolism, lipid metabolism, and protein synthesis and amino acid metabolism.

**Virus infection**	**Gene ID**	**Annotation**	**V^***a***^**	**NV^***b***^**	**FC^***c***^**	***q*-value**	**Direction**	**Gene ID-B^***d***^**
**ENERGY METABOLISM**								
TYLCV	BTA007709.1	ATP synthase gamma chain	612.89	921.90	−0.52	0.0160	Down	Bta04620
ToCV	BTA028949.1	Beta-galactosidase	359.73	169.87	0.87	0.0083	Up	Bta05309
	BTA011369.1	Alpha-glucosidase	1623.32	970.45	0.69	0.0003	Up	Bta11975
	BTA006577.1	Alpha-glucosidase	295.44	668.78	−1.03	2.17E−05	Down	Bta08426
	BTA018902.1	Alpha-glucosidase	149.28	349.03	−0.93	0.0105	Down	Bta07764
	BTA029066.1	L-lactate dehydrogenase	47.56	126.75	−0.90	0.0441	Down	Bta04403
	BTA029698.1	Alpha-amylase	294.51	524.22	−0.71	0.0261	Down	Bta04553
	BTA007709.1	ATP synthase gamma chain	1355.42	905.28	0.54	0.0188	Up	Bta04620
	BTA006898.1	Pyruvate carboxylase	6609.41	4815.26	0.43	0.0262	Up	Bta05449
	BTA007224.1	Aconitate hydratase	2261.39	3193.00	−0.47	0.0414	Down	Bta04424
TYLCV&ToCV	BTA020543.1	Alpha-glucosidase family 31	2000.60	647.23	1.22	3.33E−05	Up	Bta06849
	BTA021845.1	Alpha-glucosidase	2023.34	1005.97	0.85	0.0029	Up	Bta03818
	BTA020828.1	Alpha-glucosidase	3566.17	1716.10	0.81	0.0378	Up	Bta07453
	BTA001223.1	Glucan endo-1,3-beta-glucosidase	282.61	84.63	1.20	0.0003	Up	Bta06115
	BTA029804.1	AAA-ATPase-like domain-containing protein	73.75	11.98	0.91	0.0426	Up	Bta10446
	BTA020850.1	Malate dehydrogenase	94.48	278.14	−1.00	0.0124	Down	Bta20007
**LIPID METABOLISM**								
TYLCV	BTA023227.1	Lipase	3647.63	1500.63	1.09	3.61*E*−10	Up	Bta06883
	BTA021906.3	Fatty acid oxidation complex subunit alpha	56.09	609.10	−2.18	1.10*E*−24	Down	Bta00757
	BTA011510.1	Lipid phosphate phosphohydrolase 1	2.53	93.52	−0.77	0.0035	Down	Bta05886
	BTA005102.1	Lipid storage droplets surface-binding protein 1	432.25	735.17	−0.63	0.0129	Down	Bta04143
ToCV	BTA020719.1	Glycerol-3-phosphate acyltransferase, putative	910.38	523.43	0.72	0.0014	Up	Bta09237
	BTA006414.1	Acetyl-CoA carboxylase, putative	3531.39	2235.29	0.62	0.0003	Up	Bta14032
	BTA021906.3	Fatty acid oxidation complex subunit alpha	75.22	598.08	−2.00	4.82E−11	Down	Bta00757
	BTA007925.1	Acyl-CoA synthetase family member 2, mitochondrial	4.08	72.52	−1.58	1.04E−05	Down	Bta14516
	BTA000223.1	Delta(24)-sterol reductase	69.00	183.77	−1.04	0.0035	Down	Bta11167
	BTA024237.1	Fatty acid synthase	41.42	113.10	−0.91	0.0427	Down	Bta07569
	BTA024999.2	Lipase member H-A	247.42	443.44	−0.73	0.0147	Down	Bta03971
	BTA005102.1	Lipid storage droplets surface-binding protein 1	328.21	721.93	−0.98	8.17E−05	Down	Bta04143
	BTA027702.1	Serine palmitoyltransferase	157.37	340.51	−0.86	0.0205	Down	Bta12350
TYLCV&ToCV	BTA021906.3	Fatty acid oxidation complex subunit alpha	136.80	645.54	−1.42	8.42E−06	Down	Bta00757
	BTA011510.1	Lipid phosphate phosphohydrolase 1	3.11	99.10	−1.12	0.0017	Down	Bta05886
**PROTEIN SYNTHESIS AND AMINO ACID METABOLISM**								
TYLCV	BTA001324.1	Methionine-tRNA ligase	107.08	15.48	1.16	2.06E−05	Up	Bta08529
	BTA029395.1	Msx2-interacting protein	934.45	508.46	0.66	0.0296	Up	Bta14735
	BTA008003.1	Branched-chain-amino-acid aminotransferase	1981.69	1330.59	0.52	0.0074	Up	Bta10673
	BTA018918.1	Methionyl-tRNA formyltransferase	951.32	634.42	0.52	0.0160	Up	Bta01802
	BTA013101.2	Glutamate synthase [NADH], amyloplastic	3773.52	2854.37	0.38	0.0319	Up	Bta06960
	BTA026974.1	Alanine aminotransferase 1	3.01	64.65	−0.81	0.0031	Down	Bta15725
	BTA019051.2	Tryptophan-tRNA ligase	2085.22	2872.14	−0.43	0.0160	Down	Bta06820
	BTA009050.1	Aminopeptidase N	3128.54	4197.32	−0.40	0.0171	Down	Bta07276
ToCV	BTA026807.1	Polycomb complex protein BMI-1	42.85	1.51	0.90	0.0213	Up	Bta09503
	BTA013962.1	Eukaryotic translation initiation factor 1A	1585.75	872.38	0.76	0.0027	Up	Bta13313
	BTA012766.1	Ribosomal protein S18	3925.47	2437.28	0.65	0.0002	Up	Bta04518
	BTA025030.1	ATP-dependent RNA helicase	14477.58	10584.86	0.43	0.0179	Up	Bta02498
	BTA027577.1	ATP-dependent RNA helicase A, putative	74.57	246.20	−1.14	0.0027	Down	Bta05044
	BTA026974.1	Alanine aminotransferase 1	8.18	63.49	−1.06	0.0111	Down	Bta15725
	BTA009050.1	Aminopeptidase N	1821.95	4121.58	−1.05	1.57*E*−06	Down	Bta07276
	BTA027461.1	Thymus-specific serine protease	463.00	1101.57	−0.97	0.0043	Down	Bta01281
	BTA012512.1	Aspartate aminotransferase	89.49	231.65	−0.95	0.0176	Down	Bta04470
	BTA004061.1	60S ribosomal protein L27a	139.97	289.81	−0.85	0.0122	Down	Bta07190
	BTA011516.1	Ribosomal protein L11	1015.61	1927.32	−0.79	0.0078	Down	Bta03518
	BTA014806.1	40S ribosomal protein S17	428.91	737.17	−0.70	0.0077	Down	Bta00569
	BTA019051.2	Tryptophan-tRNA ligase	1690.76	2820.37	−0.70	3.73E−05	Down	Bta06820
	BTA014721.1	Xaa-Pro aminopeptidase 1	1081.78	1733.67	−0.62	0.0146	Down	Bta03997
TYLCV&ToCV	BTA004604.1	Eukaryotic translation initiation factor 2 subunit 1	326.24	38.97	1.54	2.60*E*−06	Up	Bta01070
	BTA007461.1	SNW domain-containing protein 1	139.01	33.76	1.13	0.0042	Up	Bta09073
	BTA004061.1	60S ribosomal protein L27a	748.72	312.81	0.97	0.0031	Up	Bta07190
	BTA015861.2	U4/U6.U5 tri-snRNP-associated protein 1	139.37	50.54	0.91	0.0419	Up	Bta03973
	BTA019051.2	Tryptophan-tRNA ligase	4918.92	3044.21	0.61	0.0346	Up	Bta06820
	BTA009053.1	Pre-mRNA-processing factor 19	183.82	573.58	−0.99	0.0180	Down	Bta07273
	BTA026974.1	Alanine aminotransferase 1	10.53	68.53	−0.89	0.0491	Down	Bta15725

a *Read count values from virus-infected whiteflies*.

b *Read count values from non-viruliferous whiteflies*.

c *Fold change (log2 ratio) of gene expression*.

d *Mapped genes in Bemisia tabaci MEAM1 genome*.

We also identified and analyzed four genes associated with lipid metabolism in TYLCV-infected whiteflies, all of which were downregulated except lipase (Table 5). A majority of the lipid metabolism genes were downregulated in ToCV-infected whiteflies; the transcription levels of only two genes encoding glycerol-3-phosphate acyltransferase and acetyl-CoA carboxylase increased (Table 5). Similar with TYLCV-infection and ToCV-infection, two lipid metabolism related genes (fatty acid oxidation complex subunit alpha and lipid phosphate phosphohydrolase 1) were significantly downregulated in TYLCV&ToCV co-infected whiteflies (Table 5).

For protein synthesis and amino acid metabolism, five genes were upregulated and three genes were downregulated in TYLCV-infected whiteflies, while 14 genes (four upregulated and ten downregulated) were responsive to ToCV infection in *B. tabaci* MED (Table 5). A total of seven genes associated with protein synthesis and amino acid metabolism were identified in TYLCV&ToCV co-infected whiteflies (Table 5). The two genes encoding tryptophan-tRNA ligase and alanine aminotransferase, which were found in the two single virus-infected whiteflies, were also detected in TYLCV&ToCV co-infected whiteflies (Table 5), suggesting common functions in response to viral infection.

### Differentially Regulated Genes Associated With Cell Function and Other Functions

Only one gene involved in cell function, which is encoding Alpha-tubulin N-acetyltransferase, was upregulated in response to TYLCV infection (Table 6). Compared to TYLCV infection, more differentially regulated genes (three upregulated, six downregulated) associated with cell function were identified in ToCV-infected whiteflies (Table 6). In addition, two genes involved in cell functions (one upregulated, one downregulated) were altered in TYLCV&ToCV co-infected whiteflies (Table 4), while the gene *BTA030040.1* encoding condensing-2 complex subunit D3 was also differentially expressed in ToCV-infected whiteflies.

**Table 6:** Differentially regulated genes associated with cell function and other functions.

**Virus infection**	**Gene ID**	**Annotation**	**V^***a***^**	**NV^***b***^**	**FC^***c***^**	***q*-value**	**Direction**	**Gene ID-B^***d***^**
**CELL FUNCTION**								
TYLCV	BTA020985.1	Alpha-tubulin N-acetyltransferase	379.57	192.15	0.76	0.0045	Up	Bta09955
ToCV	BTA001245.1	Histone H2B	42.91	0.44	1.22	0.0007	Up	Bta02155
	BTA007206.1	THAP domain-containing protein 4	249.31	115.92	0.86	0.0193	Up	Bta02969
	BTA008661.1	Inositol-3-phosphate synthase 1-B	280.56	137.12	0.84	0.0117	Up	Bta05168
	BTA030040.1	Condensin-2 complex subunit D3	274.86	572.85	−0.93	8.48E−05	Down	Bta02114
	BTA027341.1	E3 ubiquitin-protein ligase RNF139	56.76	147.20	−0.91	0.0379	Down	Bta02577
	BTA030041.1	SAP30-binding protein	30.13	92.20	−0.93	0.0427	Down	Bta02111
	BTA001462.1	G2/mitotic-specific cyclin-B3, putative	604.60	909.87	−0.54	0.0294	Down	Bta03554
	BTA027371.1	Cyclin-A1	1211.97	1703.48	−0.46	0.0346	Down	Bta07538
	BTA002011.1	5′ nucleotidase	1765.92	2426.65	−0.43	0.0480	Down	Bta07799
TYLCV&ToCV	BTA015504.1	Gelsolin	308.71	103.03	0.98	0.0180	Up	Bta11052
	BTA030040.1	Condensin-2 complex subunit D3	305.82	618.31	−0.85	0.0033	Down	Bta02114
**OTHERS**								
TYLCV	BTA019847.1	Vitellogenin	343607.6	242741.13	0.46	0.0159	Up	Bta07852
	BTA017585.2	Vitellogenin	113734.64	88528.55	0.34	0.0159	Up	Bta11903
	BTA005969.2	Methyltransferase	240.90	538.14	−0.94	7.79E−06	Down	Bta20014
ToCV	BTA017585.2	Vitellogenin	133350.16	86931.65	0.60	2.90E−06	Up	Bta11903
	BTA005969.2	Methyltransferase	122.19	528.44	−1.77	3.53E−14	Down	Bta20014
	BTA008678.3	Juvenile hormone-inducible protein 26-like protein	18.42	74.00	−0.94	0.0414	Down	Bta10000
	BTA023744.1	Juvenile hormone-inducible protein	73.59	173.61	−0.93	0.0123	Down	Bta00804
	BTA004540.1	Aldo/keto reductase	50.08	127.77	−0.92	0.0270	Down	Bta10339
TYLCV&ToCV	BTA017585.2	Vitellogenin	142603.78	93831.08	0.56	0.0060	Up	Bta11903
	BTA004564.1	Follicle cell protein 3C-1	440.15	798.59	−0.75	0.0058	Down	Bta14365
	BTA005969.2	Methyltransferase	292.28	570.38	−0.81	0.0054	Down	Bta20014

a *Read count values from virus-infected whiteflies*.

b *Read count values from non-viruliferous whiteflies*.

c *Fold change (log2 ratio) of gene expression*.

d *Mapped genes in Bemisia tabaci MEAM1 genome*.

In response to TYLCV infection, ToCV infection and TYLCV&ToCV co-infection, the vitellogenin genes were all upregulated in whiteflies (Table 6). We also detected that two genes encoding juvenile hormone-inducible proteins (Table 6), exhibited the downregulated transcription profiles with ToCV infection. The follicle cell protein, which is also associated with reproduction, was identified in TYLCV&ToCV co-infected whiteflies (Table 6). Moreover, our analysis showed both single infection with TYLCV or ToCV and TYLCV & ToCV co-infection could reduce the transcription level of methyltransferase (Table 6).

### Differentially Regulated Genes Associated With Unknown Protein

Among the TYLCV- and ToCV-responsive DEGs, seven and ten genes' functions were still unknown, while the comment gene *BTA023229.1* showed opposite regulation between these two viruses infection (Supplementary Table S2). Seven unknown proteins were differentially expressed in TYLCV&ToCV co-infected whiteflies, with three upregulated and four downregulated, while the gene *BTA027611.2* was similarly downregulated in ToCV-infected whiteflies (Supplementary Table S2).

### GO and KEGG Enrichment Analysis of DEGs

**Figure 2:**
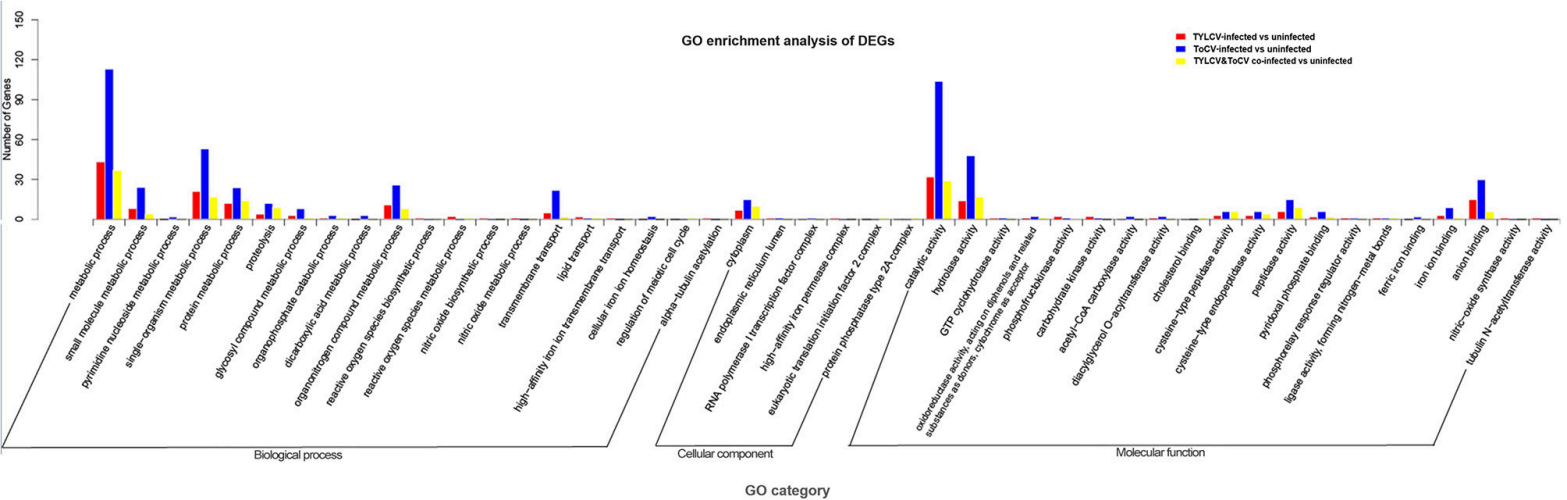
GO enrichment analysis of DEGs in *Bemisia tabaci* MED in response to TYLCV infection, ToCV infection, and TYLCV&ToCV co-infection. Bars represent the number of DEGs classified into 46 GO terms under three main categories (biological process, cellular component, and molecular function).

GO assignment was performed to classify the functions of the DEGs in response to viral infection. There were 60, 175, and 46 genes among DEGs in response to TYLCV infection, ToCV infection and TYLCV&ToCV co-infection, respectively. These were categorized under biological process, cellular component, and molecular function categories, respectively (Figure 2; Supplementary Table S3). Under the biological process category, metabolic process, and single-organism metabolic process represented the most abundant subcategories in TYLCV-infected, ToCV-infected, and TYLCV&ToCV co-infected whiteflies. The third most represented GO terms in TYLCV-infected and TYLCV&ToCV co-infected whiteflies were both protein metabolic process, while organonitrogen compound metabolic process was present as the third largest group in ToCV-infected whiteflies. Fewer DEGs were enriched in the cellular component category, and cytoplasm was the largest subcategory among the three types of viruliferous whiteflies. In TYLCV- and ToCV-infected whiteflies, catalytic activity, hydrolase activity, and anion binding were the most abundant molecular function categories. For whiteflies exposed to TYLCV&ToCV, the DEGs were significantly enriched in catalytic activity, hydrolase activity, and peptidase activity under the molecular function category.

**Figure 3:**
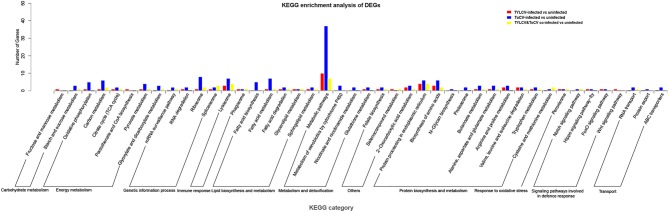
KEGG pathways enrichment analysis of DEGs in *Bemisia tabaci* MED in response to TYLCV infection, ToCV infection, and TYLCV&ToCV co-infection. Bars represent the number of DEGs classified into 44 KEGG terms under 11 main categories (metabolism and detoxification, response to oxidative stress, transport, signaling pathways involved in defense response, immune response, genetic information process, carbohydrate metabolism, protein biosynthesis and metabolism, lipid biosynthesis and metabolism, energy metabolism, and others).

Moreover, we found that 57, 184, and 44 genes among TYLCV, ToCV, and TYLCV&ToCV responsive genes, respectively, were mapped to the KEGG database and classified into 11 categories (Figure 3; Supplementary Table S4). The terms of the four most represented pathways in whiteflies fed on TYLCV were: metabolic pathways (10), protein processing in the endoplasmic reticulum (4), lysosome (3), and biosynthesis of amino acids (3). In ToCV-infected whiteflies, the highest number of genes also belonged to metabolic pathways (37), followed by ribosome (8), lysosome (7), and fatty acid metabolism (7). Compared to non-viruliferous whiteflies, the four terms of pathways most represented in TYLCV&ToCV co-infected whiteflies were metabolic pathways (7), lysosome (4), protein processing in the endoplasmic reticulum (4), and spliceosome (3).

### Validation of DEGs by RT-qPCR

**Figure 4:**
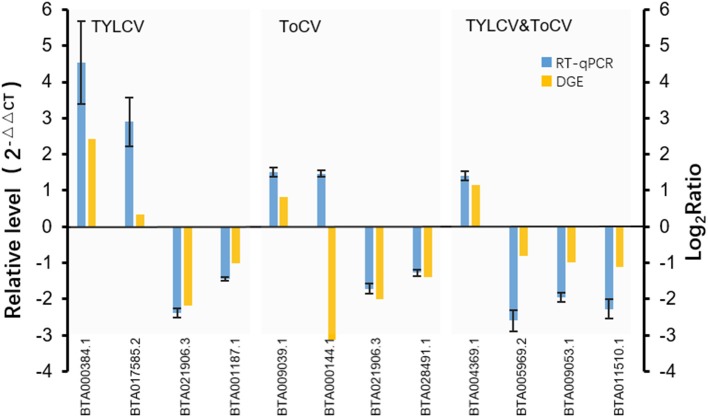
Verification of DEGs by RT-qPCR. Expression levels of 11 selected genes were measured by RT-qPCR using the 2^−ΔΔ*CT*^ method. The 11 selected genes contained nine annotated genes (*BTA017585.2*, vitellogenin; *BTA021906.3*, fatty acid oxidation complex subunit alpha; *BTA001187.1*, transmembrane protein; *BTA009039.1*, P450; *BTA000144.1*, cathepsin B; *BTA028491.1*, facilitated glucose transporter protein 1; *BTA005969.2*, methyltransferase; *BTA009053.1*, pre-mRNA-processing factor 19; *BTA011510.1*, lipid phosphate phosphohydrolase 1), and two genes encoding unknown proteins (*BTA000384.1, BTA004369.1*). The *X* axis represents the gene ID numbers of the 11 selected genes; the left *Y* axis represents the relative gene expression levels measured by RT-qPCR, while the right *Y* axis represents the log_2_Ratio of the genes resulting from DGE.

To validate the data from the DGE analyses, RT-qPCR was conducted on 11 randomly selected differentially expressed genes (Figure 4). We analyzed the transcription profiles of nine annotated genes (*BTA017585.2*, vitellogenin; *BTA021906.3*, fatty acid oxidation complex subunit alpha; *BTA001187.1*, transmembrane protein; *BTA009039.1*, P450; *BTA000144.1*, cathepsin B; *BTA028491.1*, facilitated glucose transporter protein 1; *BTA005969.2*, methyltransferase; *BTA009053.1*, pre-mRNA-processing factor 19; *BTA011510.1*, lipid phosphate phosphohydrolase 1), and two genes encoding unknown proteins (*BTA000384.1* and *BTA004369.1*). The results showed that 10 of the selected genes exhibited concordant expression patterns for both RT-qPCR and DGE (Figure 4). However, the expression trend of one gene (*BTA000144.1*) was inconsistent between RT-qPCR and DGE (Figure 4), which might have been due to the sensitivity of biases existing between the two methods. Nevertheless, the high consistency between the transcription profiles obtained by RT-qPCR and DGE confirmed the reliability of our DGE results.

## Discussion

Several transcriptome studies on whiteflies responding to TYLCV or ToCV infection have been conducted, and these have provided a large amount of valuable information on the interaction among tomato viruses, whiteflies, and plants (Kaur et al., 2017; Geng et al., 2018; Hasegawa et al., 2018). However, these studies mainly focused on infection by a single virus and were conducted on *B. tabaci* MEAM1. Thus, in the present study, we performed transcriptome analyses of *B. tabaci* MED after the whiteflies had fed on TYLCV-infected, ToCV-infected, TYLCV&ToCV co-infected, and uninfected tomatoes for 24 h to compare the gene expression pattern differences between whiteflies infected by a single virus and by both viruses. A total of 265,433,932 clean reads were obtained from eight cDNA libraries, with an average mapping rate of 81.85% to the whitefly (*B. tabaci* MED) reference genome (Xie et al., 2017). Comparative transcriptome analyses identified 359 DEGs between viruliferous and non-viruliferous whiteflies. The number of DEGs in response to ToCV infection (221 genes) was greater than that in response to TYLCV infection (78 genes). This was in accordance with previous studies with MEAM1, wherein more genes were differentially expressed on acquisition for 24 h with ToCV than TYLCV (Kaur et al., 2017; Hasegawa et al., 2018). Only 60 DEGs were detected in whiteflies feeding on TYLCV&ToCV co-infected tomato plants compared to whiteflies feeding on uninfected tomatoes. The number of TYLCV&ToCV-responsive DEGs was clearly less than those of TYLCV or ToCV, and this may be related to the complex interactions between the two viruses in the insect vectors or in the plants.

Detoxification enzymes of insects, including P450s, CarEs, UGTs, and ABCs, are important in the metabolism of xenobiotics, such as plant allelochemicals and insecticides (Scott et al., 1998; Ferry et al., 2004; Despres et al., 2007; Dermauw and Van Leeuwen, 2014). More genes associated with detoxification were regulated in ToCV-infected whiteflies than in TYLCV-infected and TYLCV&ToCV co-infected whiteflies. P450s were the main detoxification enzymes among the DEGs in virus-infected whiteflies, especially in TYLCV-infected and TYLCV&ToCV co-infected whiteflies. Furthermore, most of P450 genes were downregulated in response to the virus infection (2 of 3 genes for TYLCV infection, 4 of 5 genes for ToCV infection, 1 of 1 gene for TYLCV&ToCV co-infection). Four downregulated UGT genes were detected in ToCV-infected whiteflies, indicating that the glucuronidation of plant toxins might be inhibited by ToCV infection. Moreover, we found that the majority of detoxification enzyme genes were downregulated in the virus-infected whiteflies, which was consistent with a DGE analysis of *B. tabaci* MEAM1 in response to TYLCCNV infection (Luan et al., 2013). Whitefly susceptibility to insecticides might therefore be altered by virus infection. ABC transporters are mainly involved in the transport of xenobiotics/plant allelochemicals in arthropods (Dermauw and Van Leeuwen, 2014). The ABC gene ABCB FT/P-gp of *Drosophila melanogaster* is involved in colchicine transport (Wu et al., 1991; Tapadia and Lakhotia, 2005). Additionally, an ABC transporter with similar functions in nicotine excretion was identified in the tobacco hornworm *Manduca sexta* (Murray et al., 1994; Gaertner et al., 1998; Govind et al., 2010). It is noteworthy that two ABC genes identified in ToCV-infected whiteflies were significantly upregulated. These results suggest that improving the transport efficiency might be a whitefly strategy for coping with the plant toxins induced by viral infection.

The alpha-crystallin B chain-like proteins belonging to the small heat shock proteins (sHSP) as well as the heat shock proteins (HSP70 and HSP90) were significantly upregulated in TYLCV-infected whiteflies. The sHSPs are involved in the destabilizing effects of stressful conditions on cellular integrity (Jong et al., 1993), while HSPs are involved in preventing aggregation of denatured proteins in response to several stress factors, including oxidative, osmotic, and temperature stresses (Lindquist, 1986; Johnston et al., 1998; Sorensen et al., 2003). In addition to the HSP70 gene, we also identified the Glilial Lazarillo (GLaz) gene, a homolog of apolipoprotein D, exhibiting a positive response to the ToCV infection. This protein has been shown to play a protective role in stress situations in *D*. *melanogaster* (Sanchez et al., 2006). In contrast with the regulation of detoxification enzyme genes, most of the genes associated with stress response showed increased transcription profiles in response to either TYLCV or ToCV infection.

The innate immune system is the major weapon used by insects to fight against foreign invaders such as pathogens (Hoffmann, 1995; Irving et al., 2001). When infected by plant viruses, the cellular and humoral immune response systems are activated in insects (Luan et al., 2011; Xu et al., 2012; Shrestha et al., 2017). In the present study, higher numbers of immune response-related genes were activated in ToCV-infected whiteflies than in TYLCV-infected or TYLCV&ToCV co-infected whiteflies. Furthermore, a number of cathepsin genes were differentially expressed in whiteflies after feeding on virus-infected tomato plants. The cathepsins have been implicated in virus transmission, apoptosis, and signaling (Kubo et al., 2012; Sim et al., 2012; Saikhedkar et al., 2015). Similar to our findings, Hasegawa et al. (2018) and Kaur et al. (2017) have identified many differentially regulated genes belonging to the cathepsin B and F families in *B. tabaci* MEAM1 after feeding on TYLCV- and ToCV-infected tomatoes. Only two upregulated cathepsin B genes were found in TYLCV&ToCV co-infected whiteflies, suggesting the possible involvement of immune responses and transmission of these two viruses. Previous studies have shown that the high expression of immune genes in whiteflies with symbionts could lead to a fitness cost (Ghosh et al., 2018). Then, it can be speculated that the inducement of immune response genes may attenuate the performance of TYLCV&ToCV co-infected whiteflies on host plants. Additionally, the altered gene encoding a class B scavenger receptor, a type of surface receptor that is considered to be a regulator of phagocytosis (Franc et al., 1999; Geng et al., 2018), was found in TYLCV-infected whiteflies. Autophagy-related genes have previously been shown to be important in resistance to Begomovirus infection in whiteflies (Luan et al., 2011; Wang et al., 2016). We also identified an upregulated gene encoding sequestosome-1, which is classified as an autophagosome cargo protein, in response to TYLCV infection in whiteflies. After feeding on ToCV-infected tomato plants, the transcription level of the gene encoding the hemocyanin subunit was significantly decreased, in contrast to the transcription profile of the same gene in TYLCV-infected *B. tabaci* MEAM1 (Hasegawa et al., 2018). Hemocyanins are involved in antiviral functions in arthropods (Dolashka and Voelter, 2013), and these results suggest that the immune response modes of hemocyanins may differ according to diverse viral infections.

Our analysis identified a downregulated choline transporter-like protein gene associated with TYLCV infection. In humans, the choline transporter is a cell membrane transporter that carries choline to cholinergic neurons for acetylcholine synthesis (Okuda and Haga, 2000). The transcription level of the choline transporter-like protein was significantly decreased in ToCV-infected whiteflies, suggesting that both TYLCV and ToCV inhibit the normal transmission of neuron signals in whiteflies. Several members of the annexin group have essential roles in vesicular trafficking, adhesion, apoptosis, and viral infection (Iseki et al., 2009; Patel et al., 2011; Ma et al., 2012). For example, annexin A1 (ANXA1) was confirmed to play a detrimental role in influenza infection and positively regulated virus titers in viral infection experiments using mice (Arora, 2014). A similar role was found for salmon annexin 1 during the infection of infectious pancreatic necrosis virus (IPNV) (Hwang et al., 2007). Thus, the overexpression of the annexin gene in ToCV-infected whiteflies may contribute to the survival and transmission of the virus in vectors. The glucose transporter proteins are related to the interaction with viruses (Huang et al., 2015), and a number of facilitated glucose transporter genes were differentially regulated in ToCV-infected whiteflies *B. tabaci* MEAM1 (Kaur et al., 2017). In our study, a total of five facilitated glucose transporter genes were detected in ToCV-infected whiteflies, which suggests that these genes may participate in the interaction with ToCV *in vivo* and may be associated with virus transmission. We noticed that all four transport related genes were upregulated in TYLCV&ToCV co-infected whiteflies, while most of those genes in TYLCV- and ToCV-infected whiteflies were downregulated. The transporter proteins may exhibit positive actions, such as improving the transferring efficiency of trehalose by the upregulated facilitated trehalose transporter Tret 1 gene, in order to defend against the viral co-infection.

Most of the differentially regulated genes associated with energy metabolism encode glucosidases containing alpha-glucosidases and beta-glucosidases. Glucosidases are mainly involved in the hydrolysis of carbohydrates; they also play important roles in normal cellular function and pathogen defense (Bourne and Henrissat, 2001). Two of three alpha-glucosidase genes were downregulated in ToCV-infected whiteflies. Expression of beta-glucosidase genes in *Frankliniella occidentalis* was also depressed by TSWV infection (Zhang et al., 2013). Here, all four glucosidase genes were all upregulated in response to TYLCV&ToCV co-infection, indicating that the activation of glucosidase genes may be involved in the immune defense to co-infection with the two viruses and the complex interactions between whiteflies and viruses.

Lipid metabolism of insect vectors can be disturbed by plant viruses; this has been demonstrated in several studies and is considered to be a hallmark of cellular changes associated with viral infection (Luan et al., 2011; Xu et al., 2012; Zhang et al., 2013). Most genes in this study associated with lipid metabolism were downregulated in TYLCV-infected (3 of 4 genes) and ToCV-infected (7 of 9 genes) whiteflies, while both of the two lipid metabolism-related genes identified in TYLCV&ToCV co-infected whiteflies were downregulated. This result indicates that both single infection with TYLCV or ToCV and co-infection with TYLCV&ToCV can significantly inhibit the lipid metabolism of *B. tabaci* MED. Luan et al. (2011) also found that most genes involved in lipid metabolism were downregulated in *B. tabaci* MEAM1 when feeding on TYLCCNV-infected plants. However, other studies have shown contrasting results in TSWV-infected thrips and demonstrated that lipid metabolism in *F. occidentalis* and *F. fusca* was active in response to TSWV infection (Zhang et al., 2013; Shrestha et al., 2017).

Higher numbers of DEGs involved in protein synthesis and amino acid metabolism were identified in ToCV-infected whiteflies than in TYLCV-infected and TYLCV&ToCV co-infected whiteflies, and the majority of those genes (10 of 14 genes) were downregulated in response to ToCV infection. This suggests that protein synthesis and amino acid metabolism in whiteflies can be inhibited by ToCV infection, which is consistent with the results of previous studies on *Sogatella furcifera* and *Campoletis sonorensis* (Shelby and Webb, 1997; Xu et al., 2012). Two genes encoding eukaryotic translation initiation factors (eIF), which are involved in the initiation phase of eukaryotic translation, were upregulated in ToCV-infected and TYLCV&ToCV co-infected whiteflies. Wang et al. (2014) revealed that eIF4B of the host could inhibit influenza A virus (IAV) replication by upregulating the expression level of a key protein (interferon-induced transmembrane protein 3, IFITM3) that protects the host from virus infection. Thus, it can be inferred that the upregulation of eIF genes in viruliferous whiteflies may be a strategy in response to ToCV infection and TYLCV&ToCV co-infection. Interestingly, the two genes encoding 60S ribosomal protein L27a and tryptophan-tRNA ligase were downregulated in ToCV-infected whiteflies, but they exhibited opposite regulation profiles in TYLCV&ToCV co-infected whiteflies. The shift in gene expression may result from the alteration of insect and host plant physiologies caused by the co-infection with the two viruses.

Virus infection can lead to cellular DNA damage and the host cells will activate repair mechanisms (Huang et al., 2011). In ToCV-infected whiteflies, the histone H2B gene, which is involved in repair of DNA and regulation of transcription (Ronnigen et al., 2015), was significantly upregulated. This suggests that the histone H2B gene may help attenuate the cell damage wrought by ToCV infection. Additionally, viruses are able to alter the host cell cycle to achieve the replication and expression of their genomes, a phenomenon that has been demonstrated in geminivirus infection (Emmett et al., 2005; Ascencio-lbanez et al., 2008; Geng et al., 2018). Although ToCV cannot replicate in insect vectors, two cyclin genes (G2/motic-specific cyclin-B3 and cyclin-A1), which function in controlling the progression of cells through the cell cycle (Galderisi et al., 2003), were downregulated in ToCV-infected whiteflies. This result demonstrates that ToCV infection also disturbs the normal cell cycle in whiteflies. TYLCV&ToCV co-infection induced the expression of the gelsolin gene, an important actin regulator that is associated with the inhibition of apoptosis (Koya et al., 2000). These alterations of the cell functions caused by viruses may be an adaption or a defense strategy of insect vectors in response to viral infection.

Plant viruses can have direct and indirect effects that influence the fecundity, longevity, and survival rate of the vectors (Belliure et al., 2005; Jiu et al., 2007; Li et al., 2018). Consistent with this, our DGE analysis found several reproduction-related genes and development-related genes differentially regulated in the virus-infected whiteflies. Two vitellogenin genes were upregulated in TYLCV-infected whiteflies, supporting the result that feeding on TYLCV-infected tomato plants can increase the fecundity of *B. tabaci* MED (Su et al., 2015). Previous study showed that whiteflies MED had a shorter development time on ToCV-infected tomato plants than on healthy tomato plants (Shi et al., 2018). We also found that two juvenile hormone-inducible protein genes were both downregulated in ToCV-infected whiteflies.

GO enrichment analysis showed that the DEGs in TYLCV-infected, ToCV-infected, and TYLCV&ToCV co-infected whiteflies were all highly enriched in three functional subcategories: the metabolic process, catalytic activity, and single-organism metabolic process. Previous reports demonstrate that plant viruses can change the defense response and nutrition status of the host plants (Shi et al., 2013; Mauck et al., 2014), which might alter the expression patterns of genes involved in metabolic processes and catalytic activity. We also found several differentially regulated genes enriched in the cytoplasm, indicating that those virus-responsive genes were mainly distributed in the cytoplasm among the cellular component. Additionally, there were several DEGs classified as iron ion binding in whiteflies infected with TYLCV (three genes), ToCV (nine genes), and TYLCV&ToCV (one gene) compared with non-viruliferous whiteflies. Iron-binding proteins are vital in iron transport and sequestering iron, but an overabundance can lead to oxidative stress (Strickler-Dinglasan et al., 2006). It is also known that the major iron-binding proteins in insects play important roles in iron transport (Bartfield and Law, 1990) and immunity (Yoshiga et al., 1997, 1999). Therefore, the DEGs belonging to the iron ion binding group may participate in the defense responses to viral infection and oxidative stress.

In accordance with the results of the GO assignment, the KEGG pathway analysis showed that most DEGs in TYLCV-infected, ToCV-infected, and TYLCV&ToCV co-infected whiteflies were enriched in metabolic pathways. A majority of the genes involved in metabolic pathways were downregulated both in the TYLCV-infected (8 of 10 genes) and TYLCV&ToCV (5 of 7 genes) co-infected whiteflies. A previous study showed that 72.18% of genes enriched in primary metabolism were downregulated in TSWV-infected *F. occidentalis* (Zhang et al., 2013), which is similar to our results. However, there were 14 upregulated genes enriched in metabolic pathways in ToCV-infected whiteflies. Kaur et al. (2017) also confirmed the upregulation of metabolic pathways in ToCV-infected *B. tabaci* MEAM1. The lysosome pathway was among the most represented categories in TYLCV-infected, ToCV-infected, and TYLCV&ToCV co-infected whiteflies compared with non-viruliferous whiteflies, indicating an immune response in whiteflies during the early stages of viral infection. As expected, a number of DEGs were also enriched in the lysosome pathway in ToCV-infected *B. tabaci* MEAM1 compared with non-viruliferous whiteflies (Kaur et al., 2017), and several lysosome genes were also found to be differentially regulated in TYLCV-infected *B. tabaci* MEAM1 (Hasegawa et al., 2018). Notably, all four genes from the lysosome pathway in TYLCV&ToCV co-infected whiteflies were upregulated, while only one gene was upregulated in both TYLCV- and ToCV-infected whiteflies. We can speculate that co-infection with two viruses may induce a stronger antiviral response than single infection with either TYLCV or ToCV. Additionally, four and three genes upregulated in TYLCV infected and TYLCV&ToCV co-infected whiteflies, respectively, were significantly enriched in the pathway of protein processing in the endoplasmic reticulum, indicating that protein synthesis might be enhanced by TYLCV infection and TYLCV&ToCV co-infection.

## Conclusion

We conducted a transcriptome analysis on the whitefly *B. tabaci* MED in response to TYLCV infection, ToCV infection, and TYLCV&ToCV co-infection. *B. tabaci* MED genes responsive to viral infection were identified, including genes associated with defense response, transport, primary metabolism, cell function, and total fitness. Shifts in the expression of genes involved in transport and energy metabolism occurred between TYLCV&ToCV co-infection and single infection with TYLCV or ToCV, indicating different responses to diverse patterns of viral infection. GO and KEGG pathway enrichment analyses revealed that the metabolic process and metabolic pathways were significantly disturbed by single infection with TYLCV or ToCV as well as TYLCV&ToCV co-infection. These data increase our understanding of the whitefly-virus interaction and provide new insights into the molecular mechanisms involved in response to co-infection with different viruses. The findings may be useful for discovery of novel molecular targets that could block the spread of whitefly-transmitted viruses and help control insect vector whiteflies.

## Data Availability

The RNA-seq reads have been submitted to the SRA at NCBI under the accession PRJNA490883.

## Author Contributions

T-BD and DC conceived the study. T-BD conducted the experiments. T-BD, JL, E-HC, and J-ZN analyzed the data. T-BD drafted the manuscript. E-HC and J-ZN revised and enhanced the manuscript. All authors read and approved the final manuscript.

### Conflict of Interest Statement

The authors declare that the research was conducted in the absence of any commercial or financial relationships that could be construed as a potential conflict of interest.
