# Supplementary material for: Transcriptome Profiling of the Whitefly Bemisia tabaci MED in Response to Single Infection of Tomato yellow leaf curl virus, Tomato chlorosis virus, and Their Co-infection
Source: Front Physiol. 2019 Apr 3;10:302. doi: 10.3389/fphys.2019.00302 (PMC6457337; doi:10.3389/fphys.2019.00302)
Supplement: Supplementary file 1 [file Data_Sheet_1.zip › Supplementary Figures.DOCX]

**Supplementary Figures**

**
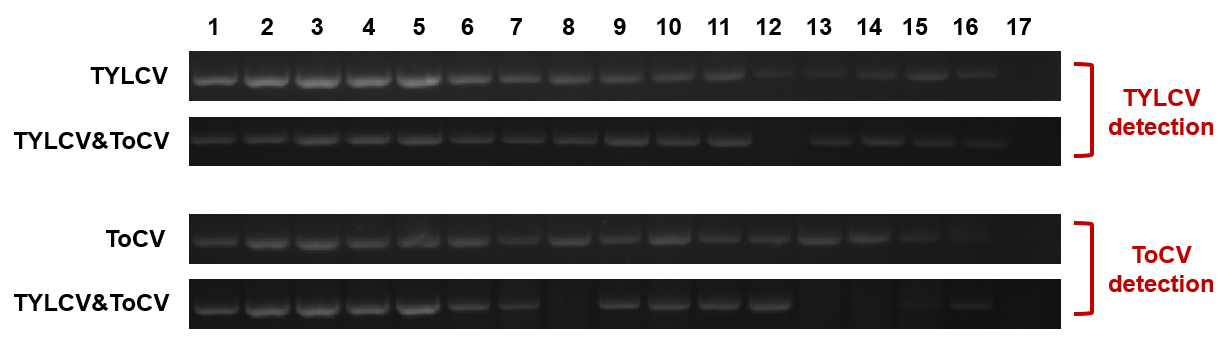
**

**Figure S1. Detection of TYLCV and ToCV from individual *Bemisia tabaci* MED using PCR.** For TYLCV detection: Lanes 1-15 indicate 15 individual whiteflies fed on TYLCV infected or TYLCV&ToCV co-infected tomato plants for 24 h; Lane 16 indicates that the individual whitefly fed on TYLCV infected tomato plants for 24 h, thus serving as the positive control; Lane 17 indicates that the individual whitefly fed on uninfected tomato plants for 24 h, thus serving as the negative control. For ToCV detection: Lanes 1-15 indicate 15 individual whiteflies fed on ToCV-infected or TYLCV&ToCV co-infected tomato plants for 24 h; Lane 16 indicates that the individual whitefly fed on ToCV infected tomato plants for 24 h, thus serving as the positive control; Lane 17 indicates that the individual whitefly fed on uninfected tomato plants for 24 h, thus serving as the negative control.


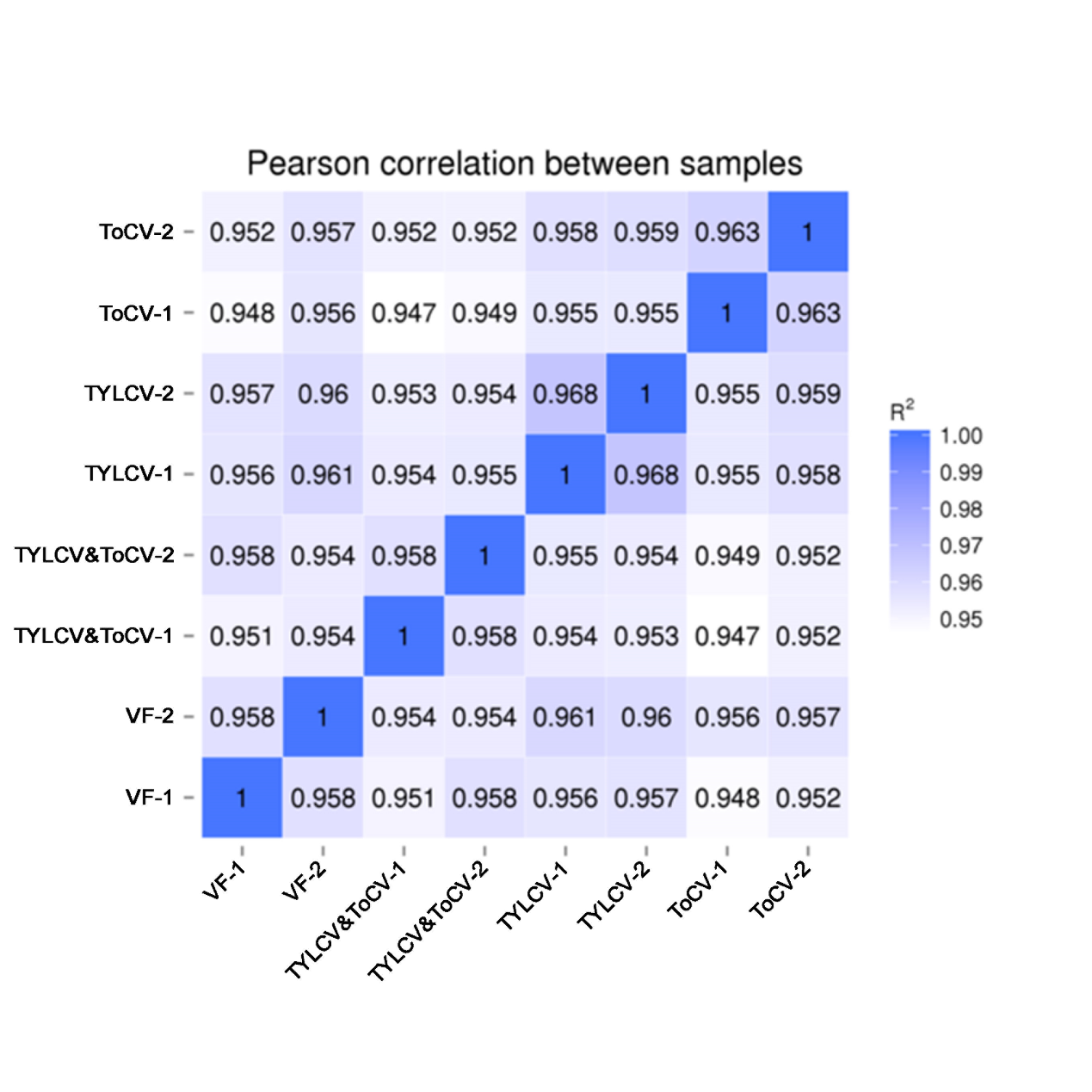
**Figure S2. Pearson correlations between eight samples.** ToCV, TYLCV, TYLCV&ToCV, and VF represent ToCV-infected whiteflies, TYLCV-infected whiteflies, TYLCV&ToCV co-infected whiteflies, and uninfected (non-viruliferous) whiteflies, respectively.
